# Psychometric validation of the Portuguese version of the Neuropathic Pain Symptoms Inventory

**DOI:** 10.1186/1477-7525-9-107

**Published:** 2011-11-30

**Authors:** Daniel Ciampi de Andrade, Karine ASL Ferreira, Carine M Nishimura, Lyn T Yeng, Abrahão F Batista, Katia de Sá, Joaci Araujo, Patrick RNAG Stump, Helena H Kaziyama, Ricardo Galhardoni, Erich T Fonoff, Gerson Ballester, Telma Zakka, Didier Bouhassira, Manoel J Teixeira

**Affiliations:** 1 Pain Center, Hospital das Clínicas, Department of Neurology of the University of São Paulo, Brazil; 2 Instituto do Câncer do Estado de São Paulo Otávio Frias de Oliveira, Brazil; 3 Divisão de Neurologia e Epidemiologia, Universidade Federal da Bahia, Brazil; 4 Coordenação de Pós-Graduação, Pesquisa e Extensão, Escola Bahiana de Medicina e Saúde Pública, Brazil; 5 INSERM U-987, CHU Ambroise Paré, APHP, F-92100 Boulogne-Billancourt, France

**Keywords:** Neuropathic Pain Symptom Inventory, Portuguese language, neuropathic pain, pain assessment, questionnaire

## Abstract

**Backgroud:**

It has been shown that different symptoms or symptom combinations of neuropathic pain (NeP) may correspond to different mechanistic backgrounds and respond differently to treatment. The Neuropathic Pain Symptom Inventory (NPSI) is able to detect distinct clusters of symptoms (i.e. dimensions) with a putative common mechanistic background. The present study described the psychometric validation of the Portuguese version (PV) of the NPSI.

**Methods:**

Patients were seen in two consecutive visits, three to four weeks apart. They were asked to: (i) rate their mean pain intensity in the last 24 hours on an 11-point (0-10) numerical scale; (ii) complete the PV-NPSI; (iii) provide the list of pain medications and doses currently in use. VAS and Global Impression of Change (GIC) were filled out in the second visit.

**Results:**

PV-NPSI underwent test-retest reliability, factor analysis, analysis of sensitivity to changes between both visits. The PV-NPSI was reliable in this setting, with a good intra-class correlation for all items. The factorial analysis showed that the PV-NPSI inventory assessed different components of neuropathic pain. Five different factors were found. The PV-NPSI was adequate to evaluate patients with neuropathic pain and to detect clusters of NeP symptoms.

**Conclusions:**

The psychometric properties of the PV-NPSI rendered it adequate to evaluate patients with both central and peripheral neuropathic pain syndromes and to detect clusters of NeP symptoms.

## Introduction

Neuropathic pain (NeP) probably concerns 7-8% of the general population [1,2]. In addition to a number of patients with various neurological diseases [3], NeP affects significant proportions of patients with diabetes [4], low back pain [5], post-surgical pain [6], cancer [7,8] and some infectious diseases [9] and has a major impact on quality of life.

Neuropathic pain syndromes are rather heterogeneous and the relationship between a certain etiology and the symptoms reported by patients are not straightforward. Different symptoms (i.e., allodynia, burning or paroxysmal pain) may coexist in the same patient and may reflect different mechanisms of disease [10]. Consistent with this hypothesis, it has been shown that different symptoms or symptom combinations may respond differently to treatment [11-13]. These data highlight the importance of a specific measurement of neuropathic pain symptoms or neuropathic components, to assess the effects of treatment both in clinical trials and in daily practice.

Only two questionnaires have been specifically developed to assess the effects of treatment in neuropathic pain syndromes [14,15]. To date, the only tool that has been validated in neuropathic pain syndromes of both central and peripheral origins is the Neuropathic Pain Symptom Inventory (NPSI). Also, it is the sole that has underwent factorial analysis confirming that the qualities of the symptoms measured by this inventory reflect distinct clusters of symptoms (i.e. dimensions) with a putative common mechanistic background [10,15].

Here we validated the translated Portuguese version of the NPSI (PV-NPSI) [16]. Portuguese is spoken by 240 million people and in the main language in more than ten countries in America, Europe, Africa and Asia [17]. So far, the NPSI has been translated into more than 60 languages, but its multidimensional structure has only been confirmed into Italian [18] and Spanish [19].

## Methods

After translation of the NPSI from the original French version and verification of its cultural and conceptual adequacy in Brazilian patients [16], the psychometric validation of the Brazilian Portuguese version of the NPSI was performed in one hundred consecutive patients with neuropathic pain seen in our outpatient pain clinic from January to July 2009. The study was approved by our I nstitution's Ethics Review Board (Hospital das Clínicas da Faculdade de Medicina da Universidade de São Paulo, São Paulo, Brazil), and written informed consent was obtained from all participants.

### Patients

Inclusion criteria were men and women with chronic (> 3 months) neuropathic pain of moderate to severe intensity (> 30 mm on a 100 mm visual analog scale) of either central or peripheral origin. Neuropathic pain was diagnosed based on the presence of pain with neuropathic characteristics in the topographic distribution of a nervous structure [20]. Lesion or disease to the somatosensory system was confirmed by nerve conduction tests, magnetic resonance imaging and blood tests when indicated. Exclusion criteria were: the presence of major depression, alcohol abuse as assessed by the CAGE questionnaire [21], the presence of an other pain of clear non neuropathic origins (e.g. myofascial pain syndrome) [22], instances where the lesion to the somatosensory system could no be clearly detected (complex regional pain syndrome) [23] and pain syndromes of clear mixed origins (failed back surgery syndrome, tumor-related pain), low level of education (less than eight years) and non Portuguese-native speakers.

### Study Design

Patients were seen in two consecutive visits, three to four weeks apart. In the first visit, before the regular consultation, they were invited to participate in the study protocol and gave their informed consent. Name, age, neuropathic pain diagnosis and associated disorders were recorded, as well as pain symptoms duration. Then they were asked to: (i) rate their mean pain intensity in the last 24 hours on an 11-point (0-10) numerical scale; (ii) complete the PV-NPSI; (iii) provide the list of pain medications and doses currently in use. Pain medication and dosing were quantified according to the Medication Quantification Score (MQS) [24]. In the second visit, patients were asked to rate the intensity of their pain on an 11-point scale, to fill out the PV-NPSI and to rate the global evolution of their pain since the first visit by the Patient Global Impression of Change (p-GIC). The evaluator also rated the global evolution of the pain by the Clinical Global Impression of Change (c-GIC). In both cases, the GIC included seven ranks ranging from 1 to 7 (1 = very much improved, 2 = moderately improved, 3 = slightly improved, 4 = no change; 5 = slightly aggravated; 6 = moderately aggravated; 7 = very much aggravated). The number of patients included in the study was calculated from the total number of items of the PV-NPSI that would undergo factorial analyses [25] and from the original NPSI publication [15].

### Assessment of the psychometrics properties of the PV-NPSI

#### Assessment of test-retest reliability

The test-retest reliability of each item and the score of the PV-NPSI was assessed using the Intraclass coefficient (ICC) calculated by the estimation of components by analysis of variance [26]. Long-term reliability was evaluated by comparing the PV-NPSI scores and sub scores in patients who did not show any change in their pain during both visits (*i.e*: score 4 - no change; on the p-CGC in the second visit).

### Factor Analysis

An exploratory factor analysis was performed using the principal component analysis as the method of extraction. The Catell Scree test was used for determining the number of factors extracted. Independent factors were obtained using the Varimax rotation method.

#### Convergent validity

Correlations between changes in pain intensity on the 11-point numeric scale and the changes in the PV-NPSI total score and sub scores were evaluated by the Spearman's correlation coefficient.

#### Analysis of sensitivity to changes between both visits

The correlation between the subjective evaluation by patients (p-GIC) in the second visit and the change in the PV-NPSI score and sub scores were assessed by the Spearman's Correlation Coefficient.

## Results

### Clinical features

Ninety-four patients were included in the study. Six failed to come to the second visit within the study interval due to personal reasons. Patient's clinical characteristics and pain etiology are expressed in Table 1.

**Table 1:** Main clinical characteristics of patients included in the study.

Clinical and demographic data	
Age	52.6 ± 14.9 (27-84)
Sex (women/men)	37/57
Mean duration of pain (months)	51.7 ± 21.4 (6-120)
Mean pain intensity (VAS)	6.7 ± 2.0 (4-10)
Mean MQS	10.1 ± 5.3 (1.0-25.0)

**Aetiology of neuropathic pain**	

Nerve trauma	15 (15.9%)
Post herpetic neuralgia	20 (21.3%)
Diabetic polyneuropathy	6 (6.4%)
Non-diabetic polyneuropathy	5 (5.3%)
Post-stroke pain	4 (4.2%)
Spinal cord trauma	9 (9.5%)
Plexus avulsion	19 (20.2%)
Trigeminal neuralgia	4 (4.25%)
Syringomyelia	2 (2.1%)
Leprosy associated neuropathic pain	10 (10.6%)

**Medication use**	

Medication Quantification Score	10.14 ± 5.96

Results are expressed in average ± standard deviation (range).

### Face validity

The PV-NPSI was filled out in less than 8 minutes by 85% of the patients. It took less than 12 minutes in the remaining. The "prevalence" (i.e. percentage of patients reporting a score > 0) in the majority of items was 65% (table 2).

**Table 2:** Frequency of items reported as > 1.

Pain Descriptor (items)	Percentage of patients who reported a score > 0
Burning	73.4%
Squeezing	57.4%
Pressure	56.3%
Electric shocks (5)	65.9%
Stabbing	47.9%
Evoked by brushing	64.8%
Evoked by pressure (8)	60.6%
Evoked by cold stimulus	63.8%
Pins and needles	68.0%
Tingling	81.9%

### Test-retest validity

Thirty patients did not present any change in their pain between both visits (ie. p-GIC). The NPSI scores of these patients were retained to evaluate the test-retest reliability of the PV-NPSI (table 3).

**Table 3:** Interclass Correlation Coefficient between of each PV-NPSI item in both visits.

	Test-retest reliability
Burning	0.9294
Pressure	0.9450
Squeezing	0.9664
Electric shocks	0.9309
Stabbing	0.9365
Pain evoked by brushing	0.6633
Pin evoked by pressure	0.7844
Pain evoked by cold stimuli	0.7820
Pins and needles	0.7596
Tingling	0.6280

Total Score	0.7678

### Factor analysis

The factor analysis identified a five-factor solution, which accounted for 71% of the total variance. Most items had high loadings on only one factor (Table 4).

**Table 4:** Rotated factor loadings and communalities: Varimax Rotation.

Variable	Factor1	Factor 2	Factor 3	Factor 4	Factor 5	Communality
Q1	-0.083	0.032	0.037	-0.929	0.050	0.875

Q2	0.538	-0.165	-0.510	-0.369	0.061	0.716

Q3	0.607	0.226	-0.427	0.091	-0.370	0.747

Q5	0.229	0.145	-0.200	-0.066	0.831	0.809

Q6	0.324	0.774	0.048	-0.108	-0.042	0.721

Q8	0.776	0.095	0.155	-0.123	0.166	0.678

Q9	0.589	0.337	-0.101	0.128	0.049	0.489

Q10	0.651	0.132	0.037	0.283	0.333	0.634

Q11	0.032	0.759	-0.199	0.073	0.191	0.658

Q12	-0.096	0.163	-0.837	0.071	0.178	0.773

Variance	2.2054	1.4420	1.2622	1.1451	1.0447	7.0994

% Var	0.221	0.144	0.126	0.115	0.104	0.710

Factor 1	Q2	Q3	Q8	Q9	Q10	

Factor 2	Q6	Q11				

Factor 3	Q12					

Factor 4	Q1					

Factor 5	Q5					

Each of the five factors corresponded to a relevant clinical component of neuropathic pain. Factor 1 included the three items related to evoked pain (i.e. pain evoked by brushing, pressure or contact with cold) and two spontaneous pain items (squeezing and pressure). Factor 2 included two items (i.e. stabbing and pins and needles), which might correspond to the paroxysmal component of spontaneous pain. Factor 3 included tingling (corresponding to the abnormal sensations). Factor 4 included one item (burning) corresponding to superficial component of ongoing pain frequently observed in neuropathic pain syndromes. Finally, factor 5 included only one item (electric shocks) corresponded to clear paroxysmal pain.

### Convergent analysis

The total score of the questionnaire (1st and 2^nd ^visits) correlated with the numerical rating scale measured in each visit (Spearman correlation = 0.40; p < 0.0001; and 0.53; p < 0.0001; respectively). However, the change in the PV-NPSI score between both visits (PV-NPSI visit 2 - PV-NPSI visit 1) only weakly correlated with the change in the visual numeric scale between both visits (2^nd ^score - 1^st ^score) (Spearman correlation = 0.22). The change in the PV-NPSI score between both visits did not significantly correlate with pain duration or medication use (MQS).

### Sensitivity to change

**Figure 1:**
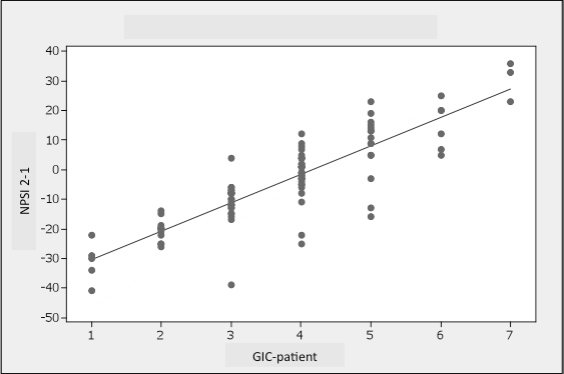
**Correlation between the GIC-p scores at the second visit and the change in the PV-NPSI score between the two visits (PV-NPSI visit 2 - PV-NPSI visit 1) (rho = 0.727)**.

The p-GIC and c-GIC scores at the second visit strongly correlated (rho = 0.727; rho = 0.645, respectively) with the change in the PV-NPSI score between the two visits (PV-NPSI visit 2 - PV-NPSI visit 1) (Figure 1).

The p-GIC and c-GIC scores at the second visit moderately correlated (rho = 0.446, 0.440) with the change in the visual numeric scale score between both visits (VNS from 2^nd ^visit - VNS from 1^st ^visit).

## Discussion

Neuropathic pain is common [27], and its prevalence in certain populations of patients is particularly high, such as in diabetics, cancer, and HIV patients [8,28]. Different screening tools have been proposed to identify patients with a higher probability to present neuropathic pain, such as the LANSS [29,30] and the DN-4 [2]. These tools have been translated and validated in different languages and are used broadly in clinical trials and epidemiological studies [7,31]. Only two scales were specifically created and validated to assess neuropathic pain syndromes [14,15]. The NPSI is the only tool validated in patients with neuropathic pain of central and peripheral origin and has a factorial design validated in a broad range neuropathic pain patients.

The present study described the psychometric validation of the Portuguese version of the NPSI. The validation process showed that the present version of the self-questionnaire is: (i) valid and reliable; (ii) it is sensitive to changes in neuropathic pain of both central and peripheral origin; and (iii) it assessed different aspects of neuropathic pain.

The PV-NPSI was filled out in a relatively short period of time making it suitable for the use in clinical practice and in clinical studies. All descriptors were reported in a significant frequency of patients, with a prevalence of 65%. We assessed the test-retest validity of the inventory in those patients who did not present any change in their pain intensity between both visits. The PV-NPSI was reliable in this setting, with a good intraclass correlation for all items.

The total score of the PV-NPSI in the 1^st ^and 2^nd ^visits correlated with the visual numeric scale score in each of these sessions. However, the change in the PV-NPSI from the 2^nd ^to the 1^st ^visit only weakly correlated to the changes in the VNS score between both instances. This is similar to what was found in the original version of the NPSI [15]. Interestingly, GIC scores in the second visit showed a high correlation with the change in the PV-NPSI between both visits, while the change in the VNS score only moderately correlated with the GIC scores. This attests that in this population of neuropathic pain patients, the total score of the PV-NPSI was better suited to assess neuropathic pain characteristics than the VNS score, showing good validity and reliability.

The factorial analysis showed that the PV-NPSI assessed different components of neuropathic pain. Five different factors were found. The first factor included evoked pain (i.e. pain evoked by brushing, pressure or contact with cold) and two spontaneous pain descriptors (squeezing and pressure). Two paroxysmal descriptors (stabbing and pins and needles) were clustered in a second factor. The three remaining descriptors were grouped in one factor each (burning pain, electric shocks and tingling). Some of the cluster patterns were slightly different from the original version where spontaneous pain and paroxysmal descriptors were clustered in a single factor each. These differences probably reflect different valences of each descriptor between the two populations [15].

Neuropathic pain is a rather heterogeneous entity and different symptoms may be caused by a single etiological factor, thus suggesting it is a "trans-etiological" entity [10]. Neuropathic pain symptoms are thought to reflect specific pain mechanisms. Two main approaches have employed questionnaires based on pain characteristics to broaden our knowledge on this topic. One used these tools to gain mechanistic insights on this pain syndrome. For example, it has been shown that the intensity of ongoing pain, as detected by the NPSI inversely correlated to the amplitude of laser evoked potentials in patients with painful distal polyneuropathy, suggesting that damage to intra-epidermal nociceptive terminals would be implicated in this specific symptom of NeP [32]. In another study, it has been shown that patients presenting exclusively with spontaneous pain according to the NPSI significantly differed from those also presenting with evoked pain. Isolated spontaneous pain was highly correlated with a greater decrease in white matter tract metrics seen under tractography, suggesting a more intense injury to the somatosensory system. Also, the presence of evoked pain in the NPSI was associated with a more discrete spinothalamic dysfunction as assessed by laser-evoked potentials when compared to patients without this pain symptom [33]. This supports the idea that different aspects of neuropathic pain as assessed by the NPSI are associated with different anatomical dysfunctions and pathophysiological backgrounds in patients with NeP. Another use of these tools was to to guide mechanism-based approaches to NeP treatment, since it has been increasingly shown that the efficacy of pharmacological treatment may vary depending on the presence of certain symptoms (mechanisms) of neuropathic pain [12,34,35].

In conclusion, the psychometric properties of the PV-NPSI render it adequate to evaluate patients with both central and peripheral neuropathic pain syndromes. The reliability of the different descriptors was adequate and sensitive to change and the NPSI may help select subgroups of NeP patients with different anatomical and mechanistic dysfunctions, and possibly different response to treatment.

## Competing interests

The authors declare that they have no competing interests.

## Authors' contributions

*Study Design: *DCA, DB, MJT. *Data Collection: *CMN, AFB, LTY, JA, PRNS, HelenaHK, KS. *Data bank feeding: *TZ, RG. *Statistical analyses: *DCA, ETF. *Manuscript writing: *DCA, KASLF. *Manuscript revising: *DB, MJT. All authors read and approved the final manuscript.
